# Evaluation of artificial intelligence (AI) chatbots for providing sexual health information: a consensus study using real-world clinical queries

**DOI:** 10.1186/s12889-025-22933-8

**Published:** 2025-05-15

**Authors:** Phyu M. Latt, Ei T. Aung, Kay Htaik, Nyi N. Soe, David Lee, Alicia J. King, Ria Fortune, Jason J. Ong, Eric P. F. Chow, Catriona S. Bradshaw, Rashidur Rahman, Matthew Deneen, Sheranne Dobinson, Claire Randall, Lei Zhang, Christopher K. Fairley

**Affiliations:** 1 Melbourne Sexual Health Centre, Alfred Health, Melbourne, Australia; 2 School of Translational Medicine, Faculty of Medicine, Nursing and Health Sciences, Monash University, Melbourne, Australia; 3 Department of Clinical Research, London School of Hygiene and Tropical Medicine, London, UK; 4 Centre for Epidemiology and Biostatistics, Melbourne School of Population and Global Health, The University of Melbourne, Melbourne, Australia; 5 Alfred Health, Melbourne, Australia; 6 Clinical Medical Research Centre, Children’s Hospital of Nanjing Medical University, Nanjing, Jiangsu Province China

## Abstract

**Introduction:**

Artificial Intelligence (AI) chatbots could potentially provide information on sensitive topics, including sexual health, to the public. However, their performance compared to nurses and across different AI chatbots, particularly in the field of sexual health, remains understudied. This study evaluated the performance of three AI chatbots - two prompt-tuned (Alice and Azure) and one standard chatbot (ChatGPT by OpenAI) - in providing sexual health information on questions that experienced sexual health nurses could correctly answer.

**Methods:**

We analysed 195 anonymised sexual health questions received by the Melbourne Sexual Health Centre phone line. A panel of experts in a blinded order using a consensus-based approach evaluated responses to these questions from nurses and the three AI chatbots. Performance was assessed based on overall correctness and five specific measures: guidance, accuracy, safety, ease of access, and provision of necessary information. We conducted subgroup analyses for clinic-specific (e.g., opening hours) and general sexual health questions and a sensitivity analysis excluding questions that Azure could not answer.

**Results:**

Alice demonstrated the highest overall correctness (85.2%; 95% confidence interval (CI), 82.1-88.0%), followed by Azure (69.3%; 95% CI, 65.3-73.0%) and ChatGPT (64.8%; 95% CI, 60.7-68.7%). Prompt-tuned chatbots outperformed the base ChatGPT across all measures. Among all outcome measures, all chatbots performed best on safety, with Azure achieving the highest safety score (97.9%; 95% CI, 96.4-98.9%), indicating the lowest risk of providing potentially harmful advice. In subgroup analysis, all chatbots performed better on general sexual health questions compared to clinic-specific queries. Sensitivity analysis showed a narrower performance gap between Alice and Azure when excluding questions Azure could not answer.

**Conclusions:**

Prompt-tuned AI chatbots demonstrated superior performance in providing sexual health information compared to base ChatGPT, with high safety scores particularly noteworthy. However, all AI chatbots showed susceptibility to generating incorrect information. These findings suggest the potential for AI chatbots as adjuncts to human healthcare providers for providing sexual health information while highlighting the need for continued refinement and human oversight. Future research should focus on larger-scale evaluations and real-world implementations.

**Supplementary Information:**

The online version contains supplementary material available at 10.1186/s12889-025-22933-8.

## Introduction

Artificial intelligence (AI) has the potential to revolutionise numerous sectors, including healthcare [1], particularly reshaping health information delivery [1–3]. AI-powered chatbots could offer a 24/7, non-judgmental and private platform for users to inquire about sensitive or stigmatised health topics, such as sexual health-related issues [4–9].

Our study focuses on generative AI, unlike traditional chatbots with pre-programmed responses [10]. Generative AIs, like ChatGPT, use natural language processing for human-like conversations [11]. They take context into account, provide personalised responses, and are more able to engage in dialogue, significantly improving the user experience [12].

Despite their potential, it is important to consider the possible downsides of AI in the delivery of healthcare information, including the risk of inaccurate or inappropriate advice that may potentially create harms [4, 13, 14]. These systems could also inadvertently retain or retrieve sensitive data. These questions raise doubts regarding the extent to which AI-powered chatbots can replace human interaction.

The Melbourne Sexual Health Centre (MSHC) receives a high volume of phone calls from clients related to sexual health concerns, which places a significant demand on the centre’s nursing staff and limits their availability for direct patient care. To address this issue, MSHC developed a text-based prompt using publicly available information from Australian guidelines for the management of sexually transmitted infections (STIs) [15] and the clinic’s website [16].

This prompt was used to create two customised chatbots through a process known as prompt engineering, which involves crafting specific instructions and providing relevant information to guide AI in generating responses tailored to a particular domain—in this case, sexual health—without modifying the underlying AI model. The chatbots developed through this process are **Alice**, based on the GPT-3.5 model and implemented on chatbotbuilder.io [17], and **Azure**, using the same GPT model on Microsoft Azure [18]. In our study, we refer to the refinement of these prompts to further tailor the chatbot responses as prompt tuning. Additionally, we included ChatGPT, developed by OpenAI, without any customisation for comparison.

Alice and Azure chatbots do not have access to or ability to request individual patient records or personal health data. This makes them valuable, low-risk resources for handling common inquiries. Our goal is to evaluate the potential use of these chatbots in handling common questions, allowing nurses to dedicate their efforts to addressing more complex patient needs requiring human attention. Early research indicated that chatbots could deliver accurate information on health topics [19–21]. However, comparative studies between AI chatbots and nurses in the context of sexual health are limited [22]. Furthermore, studies examining the performance of different AI chatbots, such as ChatGPT versus prompt-tuned chatbots, are scarce.

This study aimed to evaluate the capabilities of three different AI chatbots (Alice, Azure, and ChatGPT) compared to clinically trained human staff in responding to sexual health inquiries.

## Methods

### Study design

We conducted this cross-sectional study at the MSHC, Australia’s largest public sexual health clinic. We compared the performance of three AI chatbots against experienced sexual health nurses in responding to sexual health inquiries. We used anonymised real-world questions from the callers to the MSHC collected during routine telephone inquiries. In compliance with the Victorian Department of Health guidelines on AI use in healthcare [23], we did not directly test chatbots with clients. Instead, we designed a method that maintained standard sexual health service delivery (i.e., a telephone conversation with a nurse) while reflecting real-life queries.

### AI chatbots and prompt tuning

We configured and evaluated three AI chatbots for this study: Alice (Custom GPT-3.5-Turbo on chatbotbuilder.io), Azure (Custom GPT-3.5 on Microsoft Azure), and ChatGPT (standard OpenAI GPT-3.5). We named the chatbots based on their development platforms or origins: “Alice” for the chatbot developed on *chatbotbuilder.io* [17], “Azure” for the one implemented on Microsoft Azure, and “ChatGPT” for the unmodified OpenAI model. We will use these names consistently throughout this manuscript to refer to these specific chatbot implementations.

Table 1 provides a detailed comparison of the chatbots’ features and settings. For Alice and Azure, we employed a process known as prompt engineering to create customised chatbots [24]. This involved developing a custom set of instructions (called a “prompt”) and incorporating a specialised database of information (known as a “knowledge base”) using publicly available information from the MSHC website [16] and Australian guidelines for the management of sexually transmitted infections (STIs) [15]. This process, which we refer to as “prompt-tuning” in this study, involves designing and refining text-based prompts to optimise and tailor the chatbots’ responses to sexual health queries. While we didn’t modify the underlying AI models, we used these custom prompts and knowledge base to guide the chatbots in providing specialised sexual health information.

**Table 1:** Comparison of artificial intelligence (AI) chatbots’ features and settings

Feature	Alice (Custom GPT-3.5-Turbo)	Azure Chatbot (Custom GPT-3.5)	Base ChatGPT (OpenAI GPT-3.5)
**Platform**	chatbotbuilder.io	Microsoft Azure	OpenAI
**Model**	GPT-3.5-Turbo 16 K	GPT-3.5	GPT-3.5
**Prompt Tuning**	Yes	Yes	No
**Specialisation**	Publicly available information (MSHC website, Australian guidelines for the management of sexually transmitted infections)	Publicly available information (MSHC website, and Australian guidelines for the management of sexually transmitted infections)	Default OpenAI training data
Temperature*	0.5	0.5	Default
Maximum Tokens**	200	200	Default
**Data Privacy**	No patient data access or storage	No patient data access or storage	No patient data access or storage
**Response Limitation**	None	Unable to answer questions beyond the provided information	None

*Temperature: A parameter that controls the randomness of the AI’s outputs. A lower value (closer to 0) makes the output more focused and deterministic,* while a higher value (closer to 1) makes it more diverse and creative*

** Maximum Tokens: The maximum number of words or word pieces the AI is allowed to generate in a single response. This limits the length of the AI’s answers

The development and refinement of Alice, including iterative testing and adjustments, took approximately 4 weeks of dedicated effort from the author (PL). We initially implemented Alice on *chatbotbuilder.io* [17] and later applied the same prompt and knowledge base to create Azure on Microsoft Azure [18]. Both Alice and Azure used a default temperature setting of 0.5 (which controls the randomness of the AI’s responses) and a maximum token limit of 200 for responses (limiting the length of answers). For the Azure chatbot, we configured settings to restrict responses to information derived solely from the provided prompt and knowledge base. This configuration resulted in the chatbot acknowledging its inability to answer questions beyond the scope of the provided information. Such a restrictive setting was not available on the *chatbotbuilder.io* platform used for Alice. ChatGPT functioned as a control, utilising default OpenAI settings without customised prompt, representing a standard AI chatbot without specific sexual health training. All chatbots operate without access to or storing patient data, ensuring privacy compliance.

### Collecting the sexual health queries

Between January and April 2024, we gathered anonymised questions and responses from calls to the MSHC phone line. To reduce recall bias, sexual health nurses at MSHC documented summaries of clients’ questions and responses immediately after each routine telephone consultation. These summaries, recorded through a Microsoft Form, included the clients’ questions and the nurses’ answers while carefully excluding all identifying information.

We gathered a total of 200 question-answer pairs over the four-month period, a sample size determined to ensure a margin of error of approximately ± 7% at a 95% confidence level, balancing statistical precision with feasibility in data collection. The collected questions covered a range of sexual health topics (see Table S1 for categorisation), including STI symptoms and testing, contraception methods, clinic services and hours, and general sexual health advice. A representative example of a question with responses from nurses and the three AI chatbots is provided in Table S2.

### Preparing and processing the data

Prior to analysis, two researchers (PL and NS) verified that all summaries were free of identifiable information. We then input the summarised questions into each of the three AI chatbots: Alice, Azure, and ChatGPT. To prevent context bias, we entered each question into a new session for each chatbot. This process resulted in a total of 800 responses: 200 from each of the three chatbots, in addition to the 200 answers provided by nurses.

### Expert evaluation and consensus process

After data collection, we assembled a panel of three experts, who were both sexual health physicians and researchers, to evaluate the quality and accuracy of the responses between June and July 2024. The reviewers had between 7 and 25 years of experience in sexual health medicine (EA, KH, CKF) with extensive expertise in caring for patients seen at the study clinic. Prior to the evaluation, the research team and reviewers collaboratively defined and clarified the meaning of five key outcome measures: guidance, accuracy, safety, ease of access, and provision of only necessary information. This ensured a consistent interpretation of the criteria throughout the evaluation process. (See Table 2).

**Table 2:** Outcome Indicator definition

Outcome Measure	Definition	Options
Overall Correctness	Evaluates the factual accuracy and appropriateness of the response in addressing the specific question.	• Correct • Mostly Correct Partially Correct • Incorrect
Guidance	Assesses whether the response offers appropriate advice regarding the next steps.	• Excellent • Good • Acceptable • Poor • Very Poor
Accuracy	Assesses the factual correctness of specific information provided in the response.	
Safety	Assesses the potential risk of harm to the patient if they follow the advice given in the response. This includes potential conflict with health care providers from wrong advice.	
Ease of Understanding	Assesses the clarity and readability of the response for a general audience.	
Provision of Necessary Information Only	Assesses whether the response provides concise, relevant information without including unnecessary details that could deter the patient from using the chatbot.	

Note: The outcome measures are considered independent of each other. For example, a response can have excellent ease of understanding while inaccurate

PL developed a Qualtrics survey and conducted a pilot test with five questions and respective answers. This pilot allowed reviewers to familiarise themselves with the rating process, apply the agreed-upon definitions, and estimate the time required for the full review. In the survey, we labelled nurses’ summaries as ‘Nurses’ and assigned anonymous identifiers to the three chatbot responses. To minimise bias, we designed a blinded review process. For each question set, we consistently presented the nurse’s summary first due to its distinctive appearance (i.e., in note format rather than verbatim). The three chatbot responses followed in a randomised order, enabling blinded comparisons between the AI chatbots.

Following the pilot test, the team held a consensus meeting to discuss score discrepancies, explain reasoning based on established definitions, and reach a unified judgment. Each reviewer then independently evaluated the remaining 195 questions and answers using the agreed-upon outcome measures and rating scale.

Following individual evaluations, we conducted a final consensus process. We categorised responses into binary classifications for correctness and the five outcome measures. We identified cases where two reviewers agreed, but the third differed. In a consensus meeting, reviewers discussed these discrepancies and worked towards a unified assessment. This process ensured evaluation consistency while preserving the integrity of initial judgments.

### Statistical analysis

We used STATA (version 17, StataCorp) for data analysis in this study. To describe response lengths, we calculated the median and interquartile range (IQR) of word count for each chatbot and nurses’ responses.

For all responses, we categorised overall correctness into “correct” (combining “correct” and “mostly correct” ratings) and “incorrect” (combining “partially correct” and “incorrect” ratings). For the five outcome measures (guidance, accuracy, safety, ease of access, and provision of necessary information), we classified responses as “acceptable or better” (including “acceptable”, “good”, and “excellent” ratings) or “unacceptable” (including “poor” and “very poor” ratings).

We then calculated the proportion of correct or acceptable responses for each chatbot and nurses, using questions with correct nurse responses as the benchmark. We compared these proportions using chi-square tests, considering p-values less than 0.05 as statistically significant. We calculated 95% confidence intervals for all proportions.

We conducted subgroup analyses by stratifying questions into “General sexual health questions” and “Clinic-specific questions,” repeating our performance comparisons for each subgroup. To account for the differences in chatbot configurations, particularly Azure’s restricted response settings, we performed a sensitivity analysis to assess the impact of Azure’s restricted configuration on our overall findings and ensure a fair comparison across all chatbots. In this analysis, we reran our main analyses after excluding questions that the Azure chatbot could not answer due to its limitation to the provided prompt and knowledge base.

## Results

We analysed a total of 195 questions, following the exclusion of five questions used in pilot testing. Each question had four responses: one from a nurse and one from each of the three AI chatbots, resulting in a total of 780 responses.

**Fig. 1:**
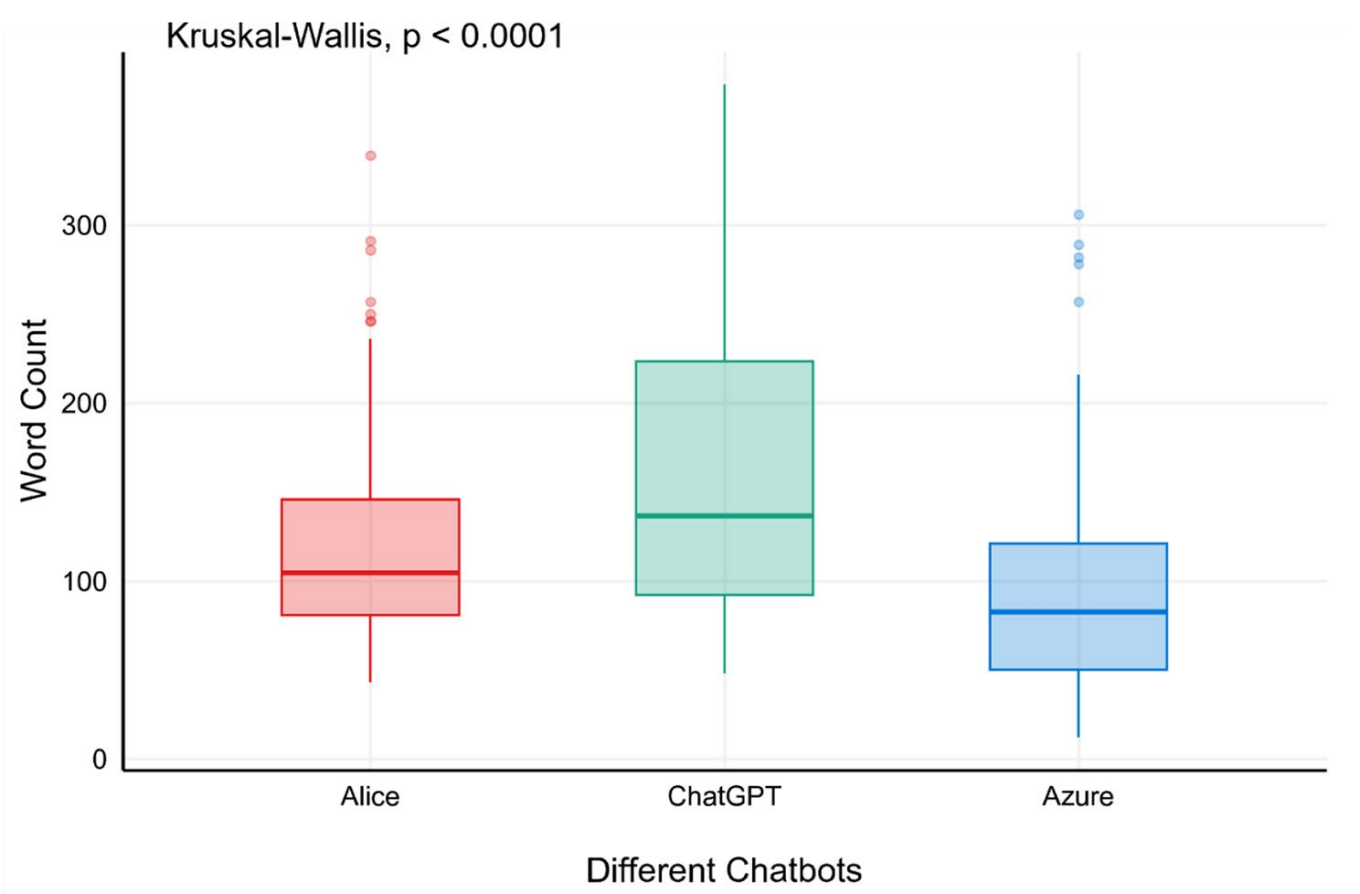
Length of Responses by Chatbots

The median length of client questions was 14 words (IQR, 10–19). Nurses’ responses were concise as they were written in the form of a summary note, with a median length of 25 words (IQR, 13–39). In contrast, AI-generated responses were substantially longer. Alice produced responses with a median length of 105 words (IQR, 81–147), ChatGPT a median of 137 words (IQR, 92–224) and Azure a median length of 83 words (IQR, 50–122) (Fig. 1).

Of the 195 questions analysed, nurses provided correct responses to 192 (98.5%) queries, which served as the benchmark. We based our analysis of chatbot performance on these 192 correctly answered questions. It’s important to note that for nurse responses, we assessed only overall correctness, whereas for chatbots, we evaluated both overall correctness and five additional outcome measures. Therefore, comparisons between nurses and chatbots are limited to overall correctness, while the five outcome measures are compared only among the three chatbots.

**Fig. 2:**
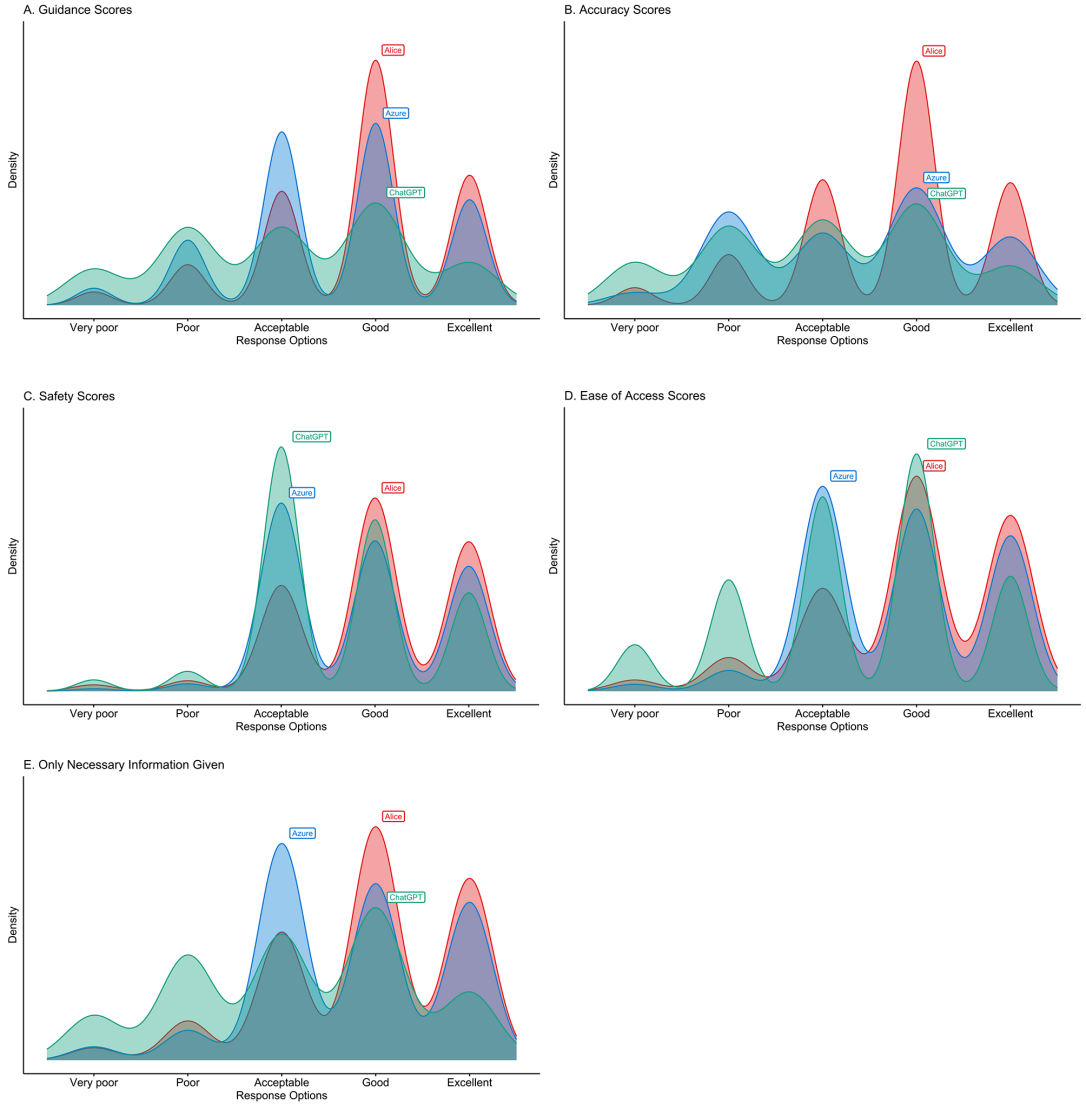
Distribution of Reviewers’ Ratings for the three Chatbots to Clients’ Questions

Alice demonstrated the highest overall correctness at 85.2% (95% CI, 82.1-88.0%), followed by Azure at 69.3% (95% CI, 65.3-73.0%), and ChatGPT at 64.8% (95% CI, 60.7-68.7%). The difference in performance between the three chatbots was statistically significant (*p* < 0.0001). Regarding the five outcome measures, Alice consistently outperformed the other chatbots in guidance (90.1%; 95% CI, 87.4-92.4%) and accuracy (87.8%; 95% CI, 84.9-90.4%). Azure showed strength in safety (97.9%; 95% CI, 96.4-98.9%) and ease of access (95.1%; 95% CI, 93.1-96.7%), outperforming Alice and ChatGPT in these areas and these findings are statistically significant with p-values less than 0.05. ChatGPT consistently showed lower scores across all measures compared to Alice and Azure (Table 3). The distribution of individual scores for each outcome measure is illustrated in Fig. 2.

**Table 3:** Overall performance of the three AI chatbots in responding to sexual health queries

	Outcome Measure	The proportion of Overall Correctness by Chatbots (*N* = 576) [192 questions]			*P*-value
		Alice	ChatGPT	Azure	
	Overall Correctness	85.2% (82.1-88.0%)	64.8% (60.7-68.7%)	69.3% (65.3-73.0%)	< 0.0001
	**Outcome Measures**	**The proportion of Acceptable and Beyond Responses by Chatbots** **(*N*** **=** **576) [192 questions]**			
**1**	**Guidance**	90.1% (87.4-92.4%)	66.1% (62.1-70.0%)	84.9% (81.7-87.7%)	< 0.0001
**2**	**Accuracy**	87.8% (84.9-90.4%)	64.9% (60.9-68.8%)	70.8% (66.9-74.5%)	< 0.0001
**3**	**Safety**	96.4% (94.5-97.7%)	94.3% (92.0-96.0%)	97.9% (96.4-98.9%)	0.005
**4**	**Ease of Access**	91.7% (89.1-93.8%)	77.6% (74.0-80.9%)	95.1% (93.1-96.7%)	< 0.0001
**5**	**Only Necessary Information Given**	91.3% (88.7-93.5%)	69.8% (65.9-73.5%)	92.7% (90.3-94.7%)	< 0.0001

### Subgroup analysis

We conducted a subgroup analysis by dividing the questions into clinic-related questions (*n* = 78) and general sexual health questions (*n* = 114). (Table S3)

For clinic-related questions, Alice demonstrated the highest overall correctness at 78.2% (95% CI, 72.4-83.3%), followed by Azure (69.2%; 95% CI, 62.9-75.1%) and ChatGPT (39.3%; 95% CI, 33.0-45.9%). Across all outcome measures for clinic-related questions, Alice and Azure consistently outperformed ChatGPT. Notably, all chatbots achieved high scores in safety, with Azure the highest (98.7%; 95% CI, 96.3-99.7%), followed by Alice (94.9%; 95% CI, 91.2-97.3%), and ChatGPT (91.0%; 95% CI, 86.6-94.4%).

For general sexual health questions, Alice demonstrated the highest overall correctness at 90.1% (95% CI, 86.4-93.0%), followed by ChatGPT at 82.2% (95% CI, 77.7-86.1%) and Azure at 69.3% (95% CI, 64.1-74.1%). Across all outcome measures for general sexual health questions, Alice consistently outperformed the other chatbots. Notably, all chatbots achieved high scores in safety, with Alice and Azure both at 97.4% (95% CI, 95.1-98.8%), followed closely by ChatGPT at 96.5% (95% CI, 94.0-98.2%).

### Sensitivity analysis

We conducted a sensitivity analysis after removing questions that Azure was unable to answer due to its restricted configuration. This analysis included 168 questions, resulting in 504 total responses across the three chatbots. (Table S4)

In this sensitivity analysis, the performance gap between Alice and Azure narrowed considerably. Alice’s overall correctness was 83.9% (95% CI, 80.4-87.0%) compared to Azure’s 79.2% (95% CI, 75.4-82.6%) (*p* = 0.051). Both chatbots showed comparable results in safety (Alice 95.8%, Azure 97.6%, *p* = 0.1) and only necessary information given (Alice 91.1%, Azure 94.4%, *p* = 0.6). Alice had higher guidance (88.7% vs. 82.7%, *p* = 0.007) and accuracy (86.3% vs. 81.0%, *p* = 0.02), while Azure had higher ease of access (94.4% vs. 91.1%, *p* = 0.04).

## Discussion

Our study revealed significant differences in performance among the three AI chatbots - Alice, Azure, and ChatGPT- in responding to sexual health questions based on actual inquiries to a sexual health clinic telephone line. The prompt-tuned chatbots, Alice and Azure, consistently outperformed the standard ChatGPT across all measures, with Alice demonstrating the highest overall correctness at 85.2%. This superior performance extended across various outcome measures, including guidance, accuracy, and provision of necessary information. Notably, all chatbots achieved high safety scores, particularly Azure reaching 97.9% in this important measure. Our subgroup analysis further highlighted the strengths of prompt-tuned chatbots, particularly in handling clinic-specific queries where Alice and Azure significantly outperformed ChatGPT. These findings align with recent research by Koh et al., who found that ChatGPT could provide helpful and accurate information regarding STIs, although they noted that the advice lacked specificity and required human oversight [25]. Our study extends these findings by demonstrating the potential benefits of prompt-tuning in enhancing AI performance for providing sexual health information. Our findings underscore the importance of continuous improvement in prompt engineering and knowledge base development for AI chatbots in healthcare applications [26, 27]. The significance of these findings lies in demonstrating that AI chatbots, especially those with domain-specific training, can provide accurate and safe sexual health information across a range of query types [28].

Our study found that prompt-tuned AI chatbots significantly outperformed the standard ChatGPT in providing sexual health information. This aligns with previous research showing the benefits of domain-specific training for AI in healthcare applications [28]. The superior performance of Alice and Azure, particularly in areas like guidance and accuracy, suggests that tailored knowledge bases and specialised prompts are crucial for AI’s effectiveness in healthcare contexts. However, we found that all AI chatbots, including the prompt-tuned chatbots, are susceptible to hallucinations - the generation of false or irrelevant information that is not grounded in the provided data or knowledge base - at some point. This issue was particularly prominent with ChatGPT, which again is in line with other studies [27, 29]. This limitation highlights the need for ongoing refinement and regular updates of AI models.

The high safety scores achieved by the chatbots, particularly Azure at 98%, is an important finding with significant implications for the potential integration of AI in healthcare settings. In this study, the reviewers paid particular attention to safety scores because previous studies have raised concerns about the safety of AI-generated health advice [30, 31], making our results particularly noteworthy. Koh et al. also found that ChatGPT provided generally safe advice for STI-related queries, although they emphasised the need for human physician involvement [25]. The fact that prompt-tuned chatbots achieved such high safety scores suggests that careful design and training might mitigate many risks associated with AI in healthcare. However, these promising results do not guarantee absolute safety in real-world applications, nor do they negate the need for human oversight. Further studies, including real-world implementations under controlled conditions, are needed to validate these findings and assess the true safety of AI chatbots in healthcare settings. Our findings demonstrate the potential for AI chatbots to serve as adjuncts to human healthcare providers, offering accessible and timely information while underscoring the importance of refinement and human oversight to ensure safe and effective implementation. It is important to note that even advice from clinical staff is not 100% safe, highlighting the complexity of healthcare information delivery.

The integration of AI chatbots for providing sexual health information raises important ethical considerations. Privacy and data security are paramount, given the sensitive nature of sexual health information [32]. Real-world implementation would require robust safeguards against data breaches and unauthorised access. Balancing AI efficiency with the irreplaceable human element in healthcare is crucial. Clear guidelines must be established for when AI should defer to human expertise, and patients should be made aware when they are interacting with an AI system rather than a human healthcare provider.

This study offers several key strengths in the methodology and design. Foremost is our use of real-world sexual health queries and responses from Australia’s largest sexual health clinic, ensuring high ecological validity and relevance to actual clinical practice. We implemented a multi-step, consensus-based review process involving expert sexual health physicians, enhancing our evaluations’ reliability and depth. Our iterative consensus-based approach differs from traditional methods that often rely on averaged scores or majority decisions. By actively addressing discrepancies and seeking unanimous agreement, we aimed to enhance the reliability and depth of our evaluations. This process allowed us to capture nuanced insights that might be lost in purely quantitative assessments and establish more definitive benchmarks for AI performance in providing sexual health information. While time-intensive, this method allows for a comprehensive exploration of evaluation criteria, potentially resulting in more robust and clinically relevant outcomes. Importantly, our study goes beyond evaluating just the ChatGPT; we included two prompt-tuned versions (Alice and Azure), providing insights into the potential of customised AI chatbots for specialised healthcare domains. This comparison of base and prompt-tuned chatbots offers valuable data on the effectiveness of domain-specific AI training. Additionally, our comprehensive assessment across five key outcome measures, including the critical aspect of safety, provides a multifaceted analysis of AI performance in a sensitive healthcare context.

Our study has several limitations. First, while our sample size of 195 questions was sufficient for our exploratory aims, a larger sample would improve the precision. Second, the data collection method, using nurse-summarised responses rather than verbatim transcripts, was chosen for feasibility and to protect patient privacy. While potentially introducing some level of recall and observer bias, this approach aligns with our goal of using nurse responses as a benchmark rather than for direct comparison with chatbots. Moreover, as the summaries were written by the nurses themselves, they are likely to accurately reflect the key points of the interactions. Third, the study’s focus on a single sexual health clinic, despite being the largest sexual health centre in Australia, may limit the diversity of queries represented. Additionally, the different operational settings of the chatbots - with Azure’s responses limited to its prompt and knowledge base, while Alice had no such restrictions - could introduce performance bias. We addressed this through sensitivity analysis, comparing performance with and without Azure’s limited responses. Lastly, the rapid evolution of AI technology means that chatbot performance may have changed since data collection, potentially affecting the long-term applicability of our results.

The findings of this study have significant implications for the future of providing sexual health and broader healthcare information. By handling routine inquiries, AI chatbots could potentially reduce the workload on healthcare professionals, allowing them to focus on the delivery of clinical care requiring human expertise. The superior performance of our prompt-tuned chatbots underscores the importance of domain-specific training in AI applications for healthcare. This implies that customised AI chatbots could be developed for various medical specialities, enhancing the quality and accessibility of health information across diverse fields. Moreover, the 24/7 availability and instant responses of AI chatbots could improve access to sexual health information, potentially enabling earlier detection and treatment of STIs. The high safety scores across two prompt-tuned chatbots are particularly encouraging, suggesting that AI could be a reliable source of health information with proper development and oversight.

Future research should expand the scope of AI chatbot evaluations in healthcare, using larger, more diverse datasets across various demographics and clinical settings. Longitudinal studies are needed to assess chatbot performance over time as AI technologies evolve. It is also important to investigate real-time patient interactions and integrate AI into hybrid care models, where AI assists human clinicians.

In conclusion, our study demonstrates the potential of prompt-tuned AI chatbots as adjuncts to human healthcare providers by offering accurate and safe sexual health information. While their consistently high safety scores are encouraging, the susceptibility to errors highlights the critical need for ongoing refinement, rigorous development, and human oversight to ensure reliable and effective integration into healthcare settings.

## Electronic supplementary material

Below is the link to the electronic supplementary material.

Supplementary Material 1

## Data Availability

The dataset supporting this study’s findings is maintained in a secure, controlled-access repository at the Melbourne Sexual Health Centre, Alfred Health due to its sensitive nature and ethical considerations. These data are not publicly available but may be obtained through a formal request process directed to the corresponding author, Dr. Phyu Mon Latt, contingent upon reasonable scientific justification and appropriate data governance assurances.

## References

[1] Alowais SA, Alghamdi SS, Alsuhebany N, Alqahtani T, Alshaya AI, Almohareb SN, et al. Revolutionizing healthcare: the role of artificial intelligence in clinical practice. BMC Med Educ. 2023;23(1):689. 37740191 10.1186/s12909-023-04698-z PMC10517477

[2] Zhang J, Oh YJ, Lange P, Yu Z, Fukuoka Y. Artificial intelligence chatbot behavior change model for designing artificial intelligence chatbots to promote physical activity and a healthy diet: viewpoint. J Med Internet Res. 2020;22(9):e22845. 32996892 10.2196/22845 PMC7557439

[3] Xiao Z, Liao V, Zhou M, Grandison T, Li Y. Powering an AI chatbot with expert sourcing to support credible health information access2023.

[4] Khawaja Z, Bélisle-Pipon JC. Your robot therapist is not your therapist: Understanding the role of AI-powered mental health chatbots. Front Digit Health. 2023;5:1278186. 38026836 10.3389/fdgth.2023.1278186 PMC10663264

[5] Wang H, Gupta S, Singhal A, Muttreja P, Singh S, Sharma P, et al. An artificial intelligence chatbot for young People’s sexual and reproductive health in India (SnehAI): instrumental case study. J Med Internet Res. 2022;24(1):e29969. 34982034 10.2196/29969 PMC8764609

[6] Nadarzynski T, Puentes V, Pawlak I, Mendes T, Montgomery I, Bayley J, et al. Barriers and facilitators to engagement with artificial intelligence (AI)-based chatbots for sexual and reproductive health advice: a qualitative analysis. Sex Health. 2021;18(5):385–93. 34782055 10.1071/SH21123

[7] Mills R, Mangone ER, Lesh N, Mohan D, Baraitser P. Chatbots to improve sexual and reproductive health: realist synthesis. J Med Internet Res. 2023;25:e46761. 37556194 10.2196/46761 PMC10448286

[8] Miklosik A, Evans N, Qureshi A. The use of chatbots in digital business transformation: A systematic literature review. IEEE Access. 2021;9:106530–9.

[9] Fan H, Han B, Gao W, Li W. How AI chatbots have reshaped the frontline interface in China: examining the role of sales–service ambidexterity and the personalization–privacy paradox. Int J Emerg Markets. 2022;ahead-of-print.

[10] Stokel-Walker C, Van Noorden R. What ChatGPT and generative AI mean for science. Nature. 2023;614(7947):214–6. 36747115 10.1038/d41586-023-00340-6

[11] Deng J, Lin Y. The Benefits and Challenges of ChatGPT: An Overview. Frontiers in Computing and Intelligent Systems. 2023.

[12] Patel AS. Docs get Clever with ChatGPT. Medscape Febr 3, 2023.

[13] Wasson EJ, Driver K, Hughes M, Bailey J. Sexual reproductive health chatbots: should we be so quick to throw artificial intelligence out with the bathwater? BMJ Sex Reproductive Health. 2021;47(1):73. 10.1136/bmjsrh-2020-200823 32883682

[14] Brown JEH, Halpern J. AI chatbots cannot replace human interactions in the pursuit of more inclusive mental healthcare. SSM - Mental Health. 2021;1:100017.

[15] Ong JJ, Bourne C, Dean JA, Ryder N, Cornelisse VJ, Murray S, et al. Australian sexually transmitted infection (STI) management guidelines for use in primary care 2022 update. Sex Health. 2023;20(1):1–8. 36356948 10.1071/SH22134

[16] Melbourne Sexual Health Centre. [Available from: https://www.mshc.org.au/

[17] AI CB. 2024 [Available from: https://www.chatbotbuilder.ai/

[18] Microsoft, Azure. AI Bot Service 2024 [Available from: https://azure.microsoft.com/en-au/products/ai-services/ai-bot-service

[19] Nadarzynski T, Bayley J, Llewellyn C, Kidsley S, Graham CA. Acceptability of artificial intelligence (AI)-enabled chatbots, video consultations and live Webchats as online platforms for sexual health advice. BMJ Sex Reprod Health. 2020;46(3):210–7. 31964779 10.1136/bmjsrh-2018-200271

[20] Potapenko I, Boberg-Ans LC, Michael, Klefter ON, Van Dijk EHC, Subhi Y. Artificial intelligence‐based chatbot patient information on common retinal diseases using < scp > chatgpt. Acta Ophthalmol. 2023. 10.1111/aos.15661 36912780

[21] Secinaro S, Calandra D, Secinaro A, Muthurangu V, Biancone P. The role of artificial intelligence in healthcare: a structured literature review. BMC Med Inf Decis Mak. 2021;21(1). 10.1186/s12911-021-01488-9 PMC8035061 33836752

[22] Ayers JW, Poliak A, Dredze M, Leas EC, Zhu Z, Kelley JB, et al. Comparing physician and artificial intelligence chatbot responses to patient questions posted to a public social media forum. JAMA Intern Med. 2023;183(6):589–96. 37115527 10.1001/jamainternmed.2023.1838 PMC10148230

[23] Victoria Department of Health. Health service use of unregulated Artificial Intelligence (AI) 2023 [Available from: https://www.safercare.vic.gov.au/sites/default/files/2023-07/Advisory%20-%20ChatGPT%20and%20Generative%20AI%20July%202023%20FINAL.pdf

[24] Lund B. The prompt engineering librarian. Libr Hi Tech News. 2023;40(8):6–8.

[25] Koh MCY, Ngiam JN, Tambyah PA, Archuleta S. ChatGPT as a tool to improve access to knowledge on sexually transmitted infections. Sex Transm Infect. 2024. 10.1136/sextrans-2024-056217 38925936

[26] Singhal K, Azizi S, Tu T, Mahdavi SS, Wei J, Chung HW, et al. Large Language models encode clinical knowledge. Nature. 2023;620(7972):172–80. 37438534 10.1038/s41586-023-06291-2 PMC10396962

[27] Kozaily E, Geagea M, Akdogan ER, Atkins J, Elshazly MB, Guglin M, et al. Accuracy and consistency of online large Language model-based artificial intelligence chat platforms in answering patients’ questions about heart failure. Int J Cardiol. 2024;408:132115. 38697402 10.1016/j.ijcard.2024.132115

[28] Martínez-Ezquerro JD. Response to: impact of ChatGPT and artificial intelligence in the contemporary medical landscape. Arch Med Res. 2023;54(5):102838. 37364482 10.1016/j.arcmed.2023.06.003

[29] Abbasian M, Khatibi E, Azimi I, Oniani D, Shakeri Hossein Abad Z, Thieme A, et al. Foundation metrics for evaluating effectiveness of healthcare conversations powered by generative AI. NPJ Digit Med. 2024;7(1):82. 38553625 10.1038/s41746-024-01074-z PMC10980701

[30] Birkun AA, Gautam A. Large Language model (LLM)-Powered chatbots fail to generate Guideline-Consistent content on resuscitation and May provide potentially harmful advice. Prehosp Disaster Med. 2023;38(6):757–63. 37927093 10.1017/S1049023X23006568

[31] Grabb D, Lamparth M, Vasan N. Risks from Language Models for Automated Mental Healthcare: Ethics and Structure for Implementation. medRxiv. 2024:2024.04.07.24305462.

[32] King AJ, Latt PM, Zhang L, Soe NN, Temple-Smith M, Maddaford K, Fairley CK, Chow EPF, Phillips TR. User experience of an AI application for predicting risk of sexually transmitted infections: A qualitative study. 2024. 10.1177/20552076241289646 PMC11489986 39430696

