# Supplementary material for: Evaluation of artificial intelligence (AI) chatbots for providing sexual health information: a consensus study using real-world clinical queries
Source: BMC Public Health. 2025 May 15;25:1788. doi: 10.1186/s12889-025-22933-8 (PMC12080182; doi:10.1186/s12889-025-22933-8)
Supplement: Supplementary file 1 — Supplementary Material 1 [file 12889_2025_22933_MOESM1_ESM.docx]

Table S1. Summary of Sexual Health Question Topics

| **No** | **Broad Category** | **Topic Category** | **Description** | **Example Questions** |
| --- | --- | --- | --- | --- |
| 1 | Clinic-specific | Clinic services and access | Questions about appointments, costs, and service availability | "Do I need a Medicare card to attend your service?" "Is MSHC working on weekends and public holidays?" |
| 2 | General Sexual Health questions | STI testing and diagnosis | Questions about testing availability, procedures, results interpretation, and symptoms | "Can I get tested for HSV as part of a routine screen?" "I have stinging when I wee and pus coming out my penis. What should I do?" |
| 3 |  | STI transmission and risk | Questions about how infections spread and risk assessment | "Can I get HIV from fingering someone?" "Can I give genital herpes to someone by using the same toilet seat?" |
| 4 |  | STI treatment & follow-up | Questions about treatment options, side effects, and post-treatment care | "What is the treatment for gonorrhea?" "I was diagnosed with chlamydia. Do I need a repeat test?" |
| 5 |  | Contraception sexual health | Questions about contraceptive options and general sexual health concerns | "Can I book an appointment to discuss my contraception options?" "Are sex toys safe? My partner wants to use them" |
| 6 |  | HIV prevention (PEP/PrEP) | Questions about HIV prevention strategies | "I had sex with someone who has HIV. Can I get PEP?" "Where can I access a doctor who will prescribe PrEP for me?" |

*Table S2. An Example Question with Nurse and Three AI Chatbot Responses*

| **Question** | **Nurses' Response** | **Alice's Response** | **ChatGPT's Response** | **Azure's Response** |
| --- | --- | --- | --- | --- |
| Do you do a Trush test for a man? | We do not test men for Thrush. | Yes, we do offer testing and treatment for thrush (Candida) in men at Melbourne Sexual Health Centre. If you have symptoms of thrush or any concerns, it's important to seek medical advice. You can visit our clinic for an assessment and appropriate management. Remember, early detection and treatment are key in addressing any health concerns. If you need further information or assistance, feel free to reach out. | I'm not capable of performing medical tests or examinations, including the "Trush test" you mentioned. It's possible you meant "Thrush test," which is a test for a fungal infection called thrush, often caused by Candida species. However, diagnosing and treating medical conditions should be done by qualified healthcare professionals such as doctors or medical specialists. If you suspect you have thrush or any other medical condition, it's important to consult with a healthcare provider for proper evaluation and treatment. | Sorry, I don't have the information to answer your question. However, the centre does offer comprehensive sexual health services, including testing and treatment for various sexually transmitted infections. It would be best to contact the centre directly or visit their website for more specific information about their services. |
| Evaluation (Reviewers' Consensus): | **Overall Correctness**: Correct | **Overall Correctness**: Incorrect **Scoring:** **Guidance**: Unacceptable **Accuracy**: Unacceptable **Safety**: Unacceptable **Ease of Access**: Unacceptable **Only Necessary Information Given**: Unacceptable | **Overall Correctness**: Correct **Scoring**: **Guidance**: Acceptable and beyond **Accuracy**: Acceptable and beyond **Safety**: Acceptable and beyond **Ease of Access**: Acceptable and beyond **Only Necessary Information Given**: Acceptable and beyond | **Overall Correctness**: Incorrect **Scoring**: **Guidance**: Unacceptable **Accuracy**: Unacceptable **Safety**: Acceptable and beyond **Ease of Access**: Acceptable and beyond **Only Necessary Information Given**: Acceptable and beyond |

Table S3. Subgroup Analysis of AI Chatbot Performance for Clinic-related and General Sexual Health Questions

| 1. **Clinic-related Questions** | | | | | |
| --- | --- | --- | --- | --- | --- |
|  | **Outcome Measure** | **The proportion of Overall Correctness by Chatbots**  **(N =234) [78 questions]** | | | **P-value** |
|  |  | **Alice** | **ChatGPT** | **Azure** |  |
|  | **Overall Correctness** | 78.2%  (72.4%-83.3%) | 39.3%  (33%-45.9%) | 69.2%  (62.9%-75.1%) | **<0.0001** |
|  | **Outcome Measures** | **The proportion of Acceptable and Beyond Responses by Chatbot**  **(N =234) [78 questions]** | | | |
| **1** | **Guidance** | 85.9%  (80.8%-90.1%) | 41.0%  (34.7%-47.6%) | 80.8%  (75.1%-85.6%) | <0.0001 |
| **2** | **Accuracy** | 83.3%  (77.9%-87.9%) | 39.3%  (33%-45.9%) | 70.5%  (64.2%-76.3%) | <0.0001 |
| **3** | **Safety** | 94.9%  (91.2%-97.3%) | 91.0%  (86.6%-94.4%) | 98.7%  (96.3%-99.7%) | 0.001 |
| **4** | **Ease of Access** | 89.7%  (85.1%-93.3%) | 69.7%  (63.3%-75.5%) | 95.3%  (91.7%-97.6%) | <0.0001 |
| **5** | **Only Necessary Information Given** | 88.9%  (84.1%-92.6%) | 58.5%  (51.9%-64.9%) | 91.5%  (87.1%-94.7%) | <0.0001 |
| 1. **General Sexual Health Questions** | | | | | |
|  | **Outcome Measure** | **The proportion of Overall Correctness by Chatbots**  **(N = 342) [114 questions]** | | | **P-value** |
|  |  | **Alice** | **ChatGPT** | **Azure** |  |
|  | **Overall Correctness** | **90.1%**  **(86.4%-93.0%)** | **82.2%**  **(77.7%-86.1%)** | **69.3%**  **(64.1%-74.1%)** | **<0.0001** |
|  | **Outcome Measures** | **The proportion of Acceptable and Beyond Responses by Chatbot**  **(N = 342) [114 questions]** | | | |
| **1** | **Guidance** | 93.0%  (89.7%-95.5%) | 83.3%  (79.0%-87.1%) | 87.7%  (83.8%-91.0%) | <0.0001 |
| **2** | **Accuracy** | 90.9%  (87.4%-93.8%) | 82.5%  (78.0%-86.3%) | 71.1%  (65.9%-75.8%) | <0.0001 |
| **3** | **Safety** | 97.4%  (95.1%-98.8%) | 96.5%  (94.0%-98.2%) | 97.4%  (95.1%-98.8%) | 0.7 |
| **4** | **Ease of Access** | 93.0%  (89.7%-95.5%) | 83.0%  (78.6%-86.9%) | 95.0%  (92.2%-97.1%) | <0.0001 |
| **5** | **Only Necessary Information Given** | 93.0%  (89.7%-95.5%) | 77.5%  (72.7%-81.8%) | 93.6%  (90.4%-95.9%) | <0.0001 |

*Table S4. Sensitivity Analysis of AI Chatbot Performance After Excluding Azure's Unable-to-answer Questions*

|  | **Outcome Measure** | **The proportion of Overall Correctness by Chatbots**  **(N = 504) [168 questions]** | | | **P-value** | **P-value (Alice Vs Azure)** |
| --- | --- | --- | --- | --- | --- | --- |
|  |  | **Alice** | **ChatGPT** | **Azure** |  |  |
|  | **Overall Correctness** | **83.9%**  **(80.4%-87.0%)** | **65.3%**  **(60.9%-69.4%)** | **79.2%**  **(75.4%-82.6%)** | **<0.0001** | **0.051** |
|  | **Outcome Measures** | **The proportion of Acceptable and Beyond Responses by Chatbots**  **(N = 504) [168 questions]** | | | |  |
| **1** | **Guidance** | 88.7%  (85.6%-91.3%) | 66.7%  (62.4%-70.8%) | 82.7%  (79.2%-85.9%) | <0.0001 | 0.007 |
| **2** | **Accuracy** | 86.3%  (83.0%-89.2%) | 65.3%  (60.9%-69.4%) | 81.0%  (77.2%-84.3%) | <0.0001 | 0.02 |
| **3** | **Safety** | 95.8%  (93.7%-97.4%) | 93.5%  (90.9%-95.5%) | 97.6%  (95.9%-98.8%) | 0.005 | 0.1 |
| **4** | **Ease of Access** | 91.1%  (88.2%-93.4%) | 79.4%  (75.6%-82.8%) | 94.4%  (92.1%-96.3%) | <0.0001 | 0.04 |
| **5** | **Only Necessary Information Given** | 91.1%  (88.2%-93.4%) | 79.4%  (75.6%-82.8%) | 94.4%  (92.1%-96.3%) | <0.0001 | 0.6 |
